# A Review of Approaches for Predicting Drug–Drug Interactions Based on Machine Learning

**DOI:** 10.3389/fphar.2021.814858

**Published:** 2022-01-28

**Authors:** Ke Han, Peigang Cao, Yu Wang, Fang Xie, Jiaqi Ma, Mengyao Yu, Jianchun Wang, Yaoqun Xu, Yu Zhang, Jie Wan

**Affiliations:** ^1^ Heilongjiang Provincial Key Laboratory of Electronic Commerce and Information Processing, School of Computer and Information Engineering, Harbin University of Commerce, Harbin, China; ^2^ College of Pharmacy, Harbin University of Commerce, Harbin, China; ^3^ Beidahuang Industry Group General Hospital, Harbin, China; ^4^ Laboratory for Space Environment and Physical Sciences, Harbin Institute of Technology, Harbin, China

**Keywords:** machine learning, drug-drug interactions, similarity, network diffusion, prediction

## Abstract

Drug–drug interactions play a vital role in drug research. However, they may also cause adverse reactions in patients, with serious consequences. Manual detection of drug–drug interactions is time-consuming and expensive, so it is urgent to use computer methods to solve the problem. There are two ways for computers to identify drug interactions: one is to identify known drug interactions, and the other is to predict unknown drug interactions. In this paper, we review the research progress of machine learning in predicting unknown drug interactions. Among these methods, the literature-based method is special because it combines the extraction method of DDI and the prediction method of DDI. We first introduce the common databases, then briefly describe each method, and summarize the advantages and disadvantages of some prediction models. Finally, we discuss the challenges and prospects of machine learning methods in predicting drug interactions. This review aims to provide useful guidance for interested researchers to further promote bioinformatics algorithms to predict DDI.

## Introduction

Drug–drug interactions (DDI) can occur when two or more drugs are used in combination (Baxter and Preston, 2010). Such interactions may enhance or weaken the efficacy of drugs, cause adverse drug reactions (ADRs) that can even be life-threatening in severe cases (Classen et al., 1997; Agarwal et al., 2020), and cause a drug to be withdrawn from the market (Lazarou et al., 1998). According to the U.S. Centers for Disease Control and Prevention, more than 10% of people take five or more drugs at the same time. Even worse, 20% of older adults take at least 10 drugs (Hohl et al., 2001), which greatly increases the risk of ADR. With an increasing number of approved drugs, the possibility for interactions between drugs increases accordingly (Khori et al., 2011). Therefore, predicting DDI in advance is both urgent and increasingly difficult in clinical practice.

*In vivo* and *in vitro* experiments can facilitate the identification of DDI, but cannot be performed in some cases due to laboratory limitations and/or high cost (Safdari et al., 2016). Thus, it is particularly important to develop computational methods to solve problems of identifying DDI. Current computational approaches to identify DDI can be divided into two categories: 1) extraction of DDI from literature, electronic medical records, and spontaneous reports; 2) use of known DDI to predict unknown DDI.

### Extraction of DDI

A large number of DDI are contained in unstructured articles, but with the explosion of biomedical literature, it has become a huge challenge to identify useful information from the vast literature and synchronize it within drug databases (Rodríguez-Terol et al., 2009; Pathak et al., 2013). Extraction of DDI is achieved by one of two approaches: pattern-based approaches and characteristics-based machine learning. The current pattern-based approach is being phased out because it relies on domain knowledge to manually classify DDI. With the emergence of annotated corpus (Segura et al., 2013), the method of extracting DDI based on machine learning becomes more and more popular. Moreover, extracting DDI from unstructured text data does not provide an early warning or identify unknown DDI, while machine learning can effectively predict it in advance (Kanehisa et al., 2010; Chen et al., 2019; Song et al., 2021).

### Prediction of DDI

Only known DDI can be extracted from unstructured articles. However, if the relevant DDI can be predicted in advance before a drug is put onto the market, drugs that cannot be used in combination can be identified. These identified DDI’s can prevent many medical errors. We first divide machine learning into traditional and non-traditional categories. In traditional machine learning methods, it is divided into similarity—based method and classification—based method. There are four broad categories of non-traditional machine learning. 1) Network propagation-based approach. The network propagation-based approach can be divided into link prediction and graph embedding according to different methods of network (graph) processing. The link prediction method takes biomedical entities as nodes and their complex interactions as edges to predict unknown relationship interactions and identify false or missing interactions. The method of graph embedding is to transform the known network (graph) into a low-dimensional space through the embedding layer and retain the information of the network (graph). 2) Matrix factorization. The matrix factorization method is to decompose the known drug interaction matrix into N low-dimensional space matrices using different decomposition methods, and then recombine them to obtain the matrix predicting drug interaction. 3) Ensemble-based approaches. The ensemble-based approaches which combine various methods for predicting drug interactions with the goal of achieving better results. 4) Literature-based methods. This approach first uses NLP to extract drug interactions from unstructured data as data sets. The extracted data are then used to predict unknown drug interactions. In the following article, the first part will introduce the database frequently used in the experiment in detail. The second part introduces several methods for predicting DDI. As shown in Table 1. The third part summarizes the article and gives their own views.

**TABLE 1:** DDI prediction methods based on machine learning.

Category		Method	Description
Traditional similarity		Vilar et al. (2012), Rus-Rao Zhang et al. (2009), Gottlieb et al. (2012)	Drug A and drug B interact to produce A specific effect, and it is likely that A drug similar to drug A (or drug B) interacts with drug B (or drug A) to produce the same effect
Traditional classification		Li et al. (2015), Jian-Yu et al. (2016), Kastrin et al. (2018)	The prediction task is simulated as a binary classification problem. Drugs interaction and non-interaction pairs were used to construct classification models
Network diffusion	Link prediction	PPIN Cami et al. (2013), Yan et al. (2019), Zhang et al. (2015), Park et al. (2015), Sridhar et al. (2016)	Using drugs as nodes, and their extensive connections and interactions as edges, to predict unknown interactions. Lable propagation, recursive least squares (RSL), traversal of graph and other methods are also used for link prediction
	Graph embedding	Decagon Wu et al., 2020, Feng et al. (2020), Ma et al. (2018), Liu et al. (2019), DeepDDI Ryu et al. (2018), Lee et al. (2019), Hou et al. (2019), Yifan et al. (2020)	Transform the graph into a low-dimensional space in which the information about the structure diagram is preserved. Automatically learn node representation in low dimensional space for prediction
Matrix factorization		IPF Vilar et al. (2013), MRMF Zhang et al. (2018), Shtar et al. (2019), ISCMF Rohani et al. (2020), DDINMF Yu et al. (2018), TMFUF Shi et al. (2018)	Matrix factorization decomposes the known DDI matrix into several potential matrices constrained by collective similarity, and then reconstructs the potential matrix to obtain a new interaction matrix
Ensemble-based approach		MLKNN Zhang et al. (2017)	Combine multiple methods to predict unknown DDI.
Based on literature		Tari et al. (2010), Tatonetti et al. (2012b), Kolchinsky et al. (2013)	Firstly, statistical or text mining methods are used to extract the reasonable relationship between drugs from unstructured data sources, and then machine learning methods are used to predict the unknown drug-drug interaction from the extracted drug-drug interaction information

## Datasets

Predicting DDI requires the use of multiple characteristics of drugs and known DDI. The most commonly used databases are: DrugBank (Knox et al., 2010), SIDER (Kuhn et al., 2016), TWOSIDES (Tatonetti et al., 2012a), Kyoto Encyclopedia of Genes and Genomes (KEGG) (Kanehisa et al., 2017). Certain databases were common in the literature-based approach, such as MedlinePlus and PubChem (Kim et al., 2019). The database is described in Table 2. DrugBank contains more than 4,100 drug entries, more than 800 FDA-approved small molecule and biotech drugs, and more than 3,200 experimental drugs. In addition, more than 14,000 protein or drug target sequences were associated with these drug entries. Each drug entry contains more than 80 data fields, with half of the information dedicated to drug/chemical data and the other half dedicated to drug target or protein data. SIDER newly released version of SIDER 4 incorporates data about drugs, targets and side effects into a more complete picture of drug mechanisms of action and how they cause adverse reactions. Included 1,430 drugs, 5,880 ADRs. PubChem is an open repository of chemical structures and their biological test results. Contains 247.3 million substance descriptions, 96.5 million unique chemical structures, provided by 629 data sources from 40 countries. It also contains 237 million bioactivity test results from 1.25 million bioassays covering more than 10,000 target protein sequences. A valid database of KEGG protein pathway information. The database is used to capture drug pathways by mapping drug targets. There are currently 75 protein pathway maps. KEGG drug database has 10,979 pieces of drug related information and 501,689 pieces of DDIs relationship. TWOSIDES is a database of drug-drug interactions with side effects. The database contains 868,221 significant associations between 59,220 drug pairs and 1,301 adverse events. MedLinePlus contains 233 abstracts of biomedical articles.

**TABLE 2:** Common database used to predict drug interactions.

Database	Entities	URL	Brief description
DrugBank	Drugs, Targets, Proteins	http://www.drugbank.ca/	Contains a lot of drug information and protein or drug target information
SIDER	Drugs, ADRs	http://sideeffects.embl.de/	Adverse drug reactions of large drugs
TWOSIDES	Drugs, ADRs	http://tatonettilab.org/resources/tatonetti-stm.html	Adverse drug reactions of large drugs
PubChem	Structure	https://pubchem.ncbi.nlm.nih.gov/	An open repository of chemical structures and their biological test results
KEGG	DDIs, Proteins	http://www.kegg.jp/	Metabolic pathways of hyperlinks between metabolites and protein/enzyme information
Medline	Abstract	https://en.wikipedia.org/wiki/MEDLINE/	Contains abstracts of several biomedical articles

## Machine Learning-Based Approach

### Similarity-Based Approach

The basic concept of traditional similarity-based approaches for prediction of DDI is as follows: if drug A and drug B interact with each other to produce a specific effect, then drugs like drug A (or drug B) are likely to produce the same effect with drug B (or drug A). With regard to drug similarity, interactions between new drugs are predicted through the fusion of similar characteristics of multiple drugs (Su et al., 2019a; Zeng et al., 2019; Zeng et al., 2020a; Fu et al., 2020; Mo et al., 2020; Zhuang et al., 2020; Shaker et al., 2021).

Vilar et al. (2012) proposed a large-scale approach based on identification of molecular similarities to analyze interactions of multiple types of drugs caused by inhibition of metabolic enzymes, transporters, and even pharmacological targets. To obtain molecular similarity, the authors first collected and processed drug molecules, then represented the resulting molecular structure as a bit vector that encoded the presence or absence of molecular features, where each feature was assigned a specific location. Finally, the calculation and data representation of similarity measurement are presented. Tanimoto coefficient (TC) was used to measure molecular fingerprints. 0 indicates the greatest dissimilarity, and 1 indicates the greatest similarity.

Ferdousi et al. (2017) calculated the similarity of drug pairs using the Rus-Rao approach based on similarity measurements of 12 binary vectors. The greater the similarity, the greater the likelihood of drugs interactions. Pharmacokinetic DDI (Zhang et al., 2009) describes the process by a drug affects the absorption, distribution, metabolism, or excretion of another drug; whereas, pharmacodynamic DDI (Imming et al., 2006) involves the process by which two or more drugs affect the same receptor to cause synergistic or harmful effects. Gottlieb et al. (2012) used a logistic classifier to infer interactions between pharmacodynamics and pharmacokinetics, as well as their severity, by integrating the similarity measurements of seven different drugs and building classification characteristics.

### Classification-Based Approach

The traditional classification-based approach involves simulating the DDI prediction task as a binary classification problem. DDI pairs and non-DDI pairs are used to build classification models. For binary classification, known interactions are used as inputs, and other drug pairs may have undetected or unobserved interactions that need to be predicted. In machine learning, similar problems are generally converted to semi-supervised learning tasks (Zhao et al., 2020; Hu et al., 2021a). In the classification task, a model is often built using classifiers such as logistic regression, Bayesian, k-nearest neighbor, random forest, and support vector machines (SVM) to predict DDI.

Li et al. (2015) designed a probability ensemble approach employing a Bayesian network model and similarity algorithm to predict drug pairs from molecular and pharmacological characteristics. Jian-Yu et al. (2016) proposed a new semi-supervised fusion algorithm based on a local classification model and Dempster–Shafer evidence theory. With this approach, new DDI may be predicted based on structural and side-effect similarity (Zhao et al., 2019). Kastrin et al. (2018) treated the process of predicting DDI as a binary classification task by predicting unknown interactions of randomly selected drugs in five large DDI databases using a link-prediction technique, and enhanced the network topology characteristics using four semantic characteristics.

Similarity based on the traditional method and based on the traditional classification method are obtained to predict the unknown drug interactions between very good results, but in these methods, the characteristics of drugs and drug interactions cannot get a good integration between known, there would be no way to use the known information to fully predict drug interactions. So, we need to develop more efficient computational methods to predict unknown drug interactions.

### Network Propagation-Based Approach

The network propagation-based approach predicts unknown DDI using a network of drug structural information or a network formed on the basis of known DDI (Lotfi Shahreza et al., 2018). In biomedical research, it is common to use information available in the network (figure) to predict unknown information or interactions, such as drug–disease associations, DDI, and protein–protein interactions. To solve these problems, link-prediction and graph-embedding approaches are generally used as detailed below.

#### Link Prediction

**FIGURE 1:**
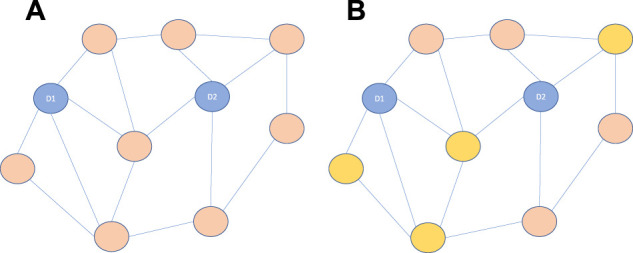
**(A)** Blue represents known interactions with the input drugs (D1, D2), and orange represents drugs whose unknown interactions need to be predicted. **(B)** Search the whole network with different methods to find the drugs most similar to D1 and D2, which are represented by yellow drugs in the figure. Finally, the possibility of interaction between yellow drugs and input drugs was predicted.

Link prediction uses biomedical entities as nodes, and their extensive connections and interactions as edges, to predict unknown interactions and identify false or missing interactions. In Figure 1. Initially, link-prediction approaches assessed the similarity between nodes based on local topological characteristics (Zhou et al., 2009). However, random walk algorithms have become more frequently used for link prediction thanks to the development of global network topology. In addition, many approaches have been employed for link prediction, such as label propagation (Zhang P et al., 2015), probability soft logic (PSL) model (Bach et al., 2015), and graph traversal algorithms (Hu et al., 2020; Hu et al., 2021b).

The similarity-based approaches mentioned above often ignore the structural information encoded in drug biological networks and their interactions. Both the network similarity-based approach and memory network reasoning approach can solve this problem. Specifically, the network similarity-based approach is presented with graphs or reasoned directly through graph structures. Yan et al. (2019) proposed a binary vector-based approach incorporating drug chemistry, biological, and phenotypic data. Briefly, comprehensive drug characteristic similarity was calculated by the isotope similarity approach, a node-based drug network diffusion approach was used to calculate the initial score of the relationship between new drugs, and new DDI were deduced using the recursive least squares (RLS) algorithm.

Cami et al. (2013) described that the DDI of unknown drugs may be predicted by building a network of known DDI. In such a network, drugs are expressed as nodes, while known interactions between known drugs are expressed as edges to predict unknown edges. The first is to integrate data from a number of different sources, including safety data, taxonomic data and data related to the intrinsic characteristics of the drug. The authors construct a network representation of all DDI, where each node represents a drug, and each connecting two nodes represents a known interaction between the two drugs. Next, the binary variables representing the presence or absence of interactions between drug I and drug J were modeled as Bernoulli random variables and three types of covariate functions. By fitting all possible univariate logistic regression models, the authors began to develop models to assess the univariate effect and significance of each covariable.

Most approaches use only first-order similarity, while label propagation considers higher-order similarity. Zhang W et al. (2015) proposed prediction of DDI by integrating clinical side effects extracted from prescription drug packages and United States Food and Drug Administration (FDA) Adverse Event Reporting System, as well as chemical structures extracted from PubChem. They also proposed a framework for comprehensive label propagation that considers similarities of higher-order interactions. First, the authors used the Jaccard index to calculate the similarity between all fingerprints. The authors then create a matrix so that the rows and columns represent the drug, and each cell represents the TC between the fingerprint and the drug. Therefore, drug information from chemical structure, prescription package inserts, and FAERS is transferred to a matrix of chemical similarity, label side effect similarity, and off-label side effect similarity. The authors then use a tag propagation algorithm. The label propagation algorithm solves the following problem: given a weighted network without direction, estimate the labels of the remaining unlabeled nodes. The author takes different drugs as nodes on the network and calculates the edge weights on the network using the drug similarity evaluated by the method in the last section. For each drug, all other drugs in the labeling network are positive, and unlabeled drugs will have DDI with this drug if they know there is a DDI associated with this drug, and use the label to spread the likelihood of this drug network estimate.

The random walk algorithm provides a powerful function to randomly select objects from adjacent nodes in the network, and then repeats this process constantly to capture information in the network. Park et al. (2015) conceived the method of predicting pharmacokinetic DDI by comparing signal propagation and protein–protein interactions in the network. To achieve this, they applied random walk and restart algorithms to simulate signal propagation from drug targets and capture the possibility of distant interference. The probability of each protein was calculated by the random walk with restart (RWR) algorithm. Protein probabilities are used to represent the effect of drug targets on the protein-protein interaction (PPI) network. The RWR algorithm simulates random walkers until the probability of all proteins on the PPI network is saturated. Next, the authors calculated the protein fraction, which represents the overlapping effects of the two drugs on the same protein. In addition, DDIScore is calculated by summing up the protein fractions of all proteins. The authors used DDIScore as a measure of the likelihood of DDI occurring between drugs.

The advantages of PSL are highly extensible and easy to extend (Bach et al., 2015). Sridhar et al. (2016) inferred DDI from the similarities of multiple drugs and networks of their known interactions, using the joint probability under the PSL framework. Importantly, this approach can be easily extended by different informants and similarities for a variety of applications.

#### Graph-Embedding Approach

**FIGURE 2:**
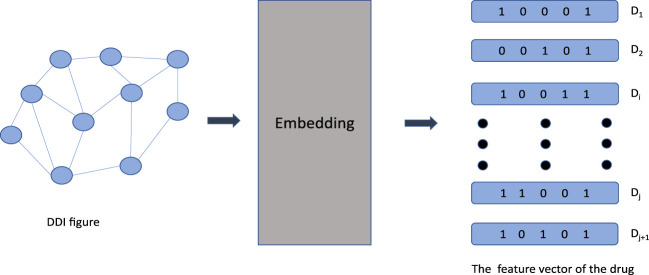
Start by creating the graph structure of the DDI. D _1_ through D _j+1_ indicates the drug number. Nodes represent drugs and edges represent relationships between drugs. The high dimensional graph structure is transformed into low dimensional vector by embedding layer.

The purpose of graph-embedding approaches is to transform a graph into a low-dimensional space in which structural information about the diagram is preserved. Nodes in the low-dimensional space of automatic learning indicate that prediction has been conducted. Notably, in the field of biology graph-embedding approaches have proven to be more effective in the field of biology than traditional approaches (Tang et al., 2015). In Figure 2. Below, graph convolution network (GCN), automated encoders and deep neural networks (DNN) used to predict DDI are described.

The GCN summarizes all the characteristic vectors of adjacent nodes in the graph by considering the characteristics of data structure extraction, and then forms a summary in the spatial domain (Wu et al., 2020). Marinka et al. (2018) built a GCN architecture (Decagon) for predicting DDI. Multi-model diagrams were constructed using protein-protein interactions, drug-protein interactions, and drug-drug interactions. By using Decagon to predict multi-relation link on multi-model graph, we can not only identify whether any two drugs interact with each other in the graph, but also determine the type of interaction. Ma et al. (2018) considered each type of drug characteristic as a view, calculated the similarity of each view, and then used a multi-view graphical automatic encoder to integrate drug similarity. Subsequently, an attention mechanism was employed to select the view, therefore improving the explanatory nature of the experiment. For modeling, each drug was a node in the drug association network, which was extended by the GCN to embed the characteristics and edges of the multi-view node.

Feng et al. (2020) Combination of GCN and DNN models has also been used to extract structural drugs from the DDI network, in order to predict DDI. DDI predictions can be solved in a three-step approach. First, the potential eigenvectors of each drug were obtained through the function F1. Then the potential vectors of the two drugs are aggregated into an eigenvector to represent the drug pair. Finally, the network is reconstructed through F2. The F1 function is called a feature extractor and the F2 function is called a predictor. Firstly, the GCN model was established to extract the latent features of low dimensional embedding of drugs from DDI network. The latent eigenvectors of the drug are then aggregated to represent drug pairs. Finally, the fused eigenvectors are fed into DNN to predict DDI.

An automatic encoder is an unsupervised neural network whose input and output errors can be minimized through an encoder and decoder. Liu et al. (2019) proposed a multi-pattern, deep autoencoder, drug expression-learning approach based on DDI prediction, which can simultaneously learn the uniform expression of the drug from its functional network. The authors integrate all four characteristics of the drug (chemical substructure, target, enzyme and pathway) to learn about drug characterization and then develop an effective model to predict drug interactions. Consider each drug data set as a view of the drug signature network, so there are five drug signature networks. We use deep neural networks to deal with representational learning. In the DDI-MDAE model, each drug signature network is trained through a deep automatic coding channel and shares the representation of the drug in the hidden layer for simultaneous learning. A unified sharing representation captures the interrelationships between different networks. On this basis, the author employs four operators to represent drug pairs and train a random forest classifier to predict potential drug-drug interactions.

Deep learning can automatically extract the characteristics of drugs from a dataset and conduct autonomous learning through a multi-layer network to predict unknown DDI. As an artificial neural network with multiple processing layers, DNN can be used to learn highly abstract expressions (Cheng et al., 2018; Su et al., 2019b; Wang et al., 2020a; Cai et al., 2020; Jia et al., 2020; Li et al., 2020; Zhu et al., 2020; Cai et al., 2021; Jin et al., 2021; Liu J et al., 2021; Liu Q et al., 2021; Su et al., 2021; Zhao et al., 2021). To predict unknown DDI, DNN-based approaches often build a framework using a DNN generated from a variety of drug data. Ryu et al. (2018) proposed that a variety of DDI can be predicted by generating a structural similarity profile of drugs that can be used as the “DeepDDI” for the prediction characteristic vector. SMILES were used to generate a feature vector called structural similarity profile (SSP) for each drug in the drug pair. The SSP is designed to effectively capture the unique structural characteristics of a given drug and correlate that characteristic with a set of reported DDI types. In order to predict the DDI type of a given drug pair, two SSPS were generated for each drug pair and combined into a single vector after dimensionality reduction. The combined SSP was the feature vector of the drug pair. A combined SSP of all DDI in a DDI dataset was created and the entire set was used to develop a DNN for accurately predicting DDI types. The author uses cross entropy as loss function and Adam optimization method to train DNN by minimizing prediction error.

Lee et al. (2019) proposed the use of three automatic encoders and a deep feed-forward network to predict DDI. Structural similarity profiles (SSP), target gene similarity profiles (TSP) and Gene Ontology (GO) term similarity profiles (GSP) were measured by autoencoder for dimension reduction (Wang et al., 2020b; Zeng et al., 2020b; Wang Y et al., 2020). The three autoencoders are all isomorphic, the size of input layer and output layer are 3194 and 600 respectively, and the size of hidden layer are 1000, 200 and 1000 respectively. The deep feedforward network has an input layer of size 600, six hidden layers of size 2000 and an output layer of size 106. Batch size is 256, the learning rate of autoencoder is 0.001, and the learning rate of feedforward network is 0.0001. The activation functions of autoencoder and feedforward network are sigmoID and ReLU. Sigmoid is used as the activation function of the output layer of the feedforward network. The number of epochs was 850, using Adam as the optimizer for the feedforward network and RMSprop as the Autoencoder optimizer. To avoid overfitting, The author uses apply dropout of 0.3 and batch normalization to feedforward networks and Autoencoders.

Hou et al. (2019) proposed the use of DNN to predict DDI, with drugs expressed as a characteristic generated by the SMILE code and entered into a DNN. Yifan et al. (2020) proposed a DDI multimodal deep-learning framework that predicts DDI event types by combining chemical substructures, targets, enzymes, and pathways with deep learning; four drug characterization vectors were calculated and put into the DNN network for training.

### Matrix Factorization-Based Approach

**FIGURE 3:**
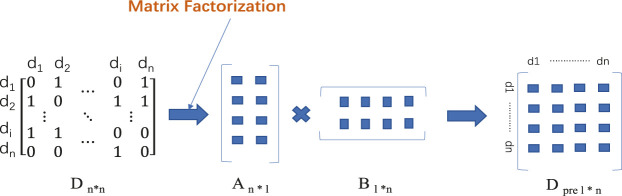
The process of matrix factorization method. Firstly, the matrix D _n*n_ of known DDI is constructed, and the matrix D is decomposed into A _n * l_ and B _l * n_ by different matrix factorization methods. Multiply the two resulting matrices, and you get the matrix D _pre l * n_ that predicts DDI.

Matrix factoring provides a mathematical basis for various modeling of the biological information problem (Martin et al., 2016). The matrix-factoring approach breaks down the DDI matrix into several matrices, extracts potential characteristics therefrom, and rebuilds the matrix to identify new DDI. In Figure 3. Traditional matrix-factoring approaches involve single-value decomposition (SVD) (Sarwar et al., 2000), non-negative matrix factoring (NMF) (Lee and Seung, 1999), and probability matrix factorization (PMF) (Mnih and Salakhutdinov, 2008). However, newly developed matrix decomposition models based on neural networks have been improved in terms of DDI performance. Although the matrix factorization method can achieve good results in predicting drug interactions, the explicability of matrix factorization method is poor and further research is needed. In the matrix factorization method, attention should also be paid to how to better integrate the characteristics of drugs as constraints of matrix decomposition.

Vilar et al. (2013) developed a new approach based on drug interaction profile fingerprinting (IPF). The IPF matrix is used to measure the similarity of drugs, and the interactive probability matrix is calculated by multiplying the DDI matrix by the IPF matrix. New DDI are predicted from the resulting interactive probability matrix. The author first generated an established database of drug interactions (matrix M1). The collected DDI set was converted into a binary matrix of 928*928, with a value of 1 indicating the interaction between the two drugs and a value of 0 indicating no interaction. Then, the similarity matrix M2 of interactive profile is generated, which is divided into three steps: the first step is IPFs calculation, the second step is fingerprint similarity calculation, and the last step is M2 construction matrix. The matrix makes rows and columns represent drugs, and each number represents contour similarity based on TC interactions between corresponding drug pairs. Finally, you can predict the new DDIs (matrix M3). Multiply matrix M1 by matrix M2 to obtain M3, and then generate a new set of predicted DDI from M3, and capture the biological effects provided by the original DDI source in M1 and associate them with the new DDI.

Rohani et al. (2020) proposed an integrated similarity constraint matrix factorization (ISCMF) to predict DDI. It can be divided into two steps: first, the integrated similarity matrix is generated as the constraint matrix of matrix decomposition, and then the matrix decomposing the matrices of known drug interactions to obtain two matrices containing potential similarity. Multiply these two matrices to get the matrix that predicts DDI. In the generation of integration similarity, eight similarity matrices were generated based on eight drug characteristics, and k optimal subsets were selected by low entropy and redundancy. Finally, Similarity matrix of K subsets was integrated by similarity network fusion (SNF) method. Based on the known drug interaction matrix, the author decomposed this matrix as the input, integrated similarity as the constraint conditions of decomposition, and finally obtained two decomposition matrices. If you multiply these two matrices, you get the matrix that predicts DDI. Because in decomposition, the loss function has multiple regularization and similarity constraints. Therefore, it can be assumed that the new interaction is a combination of known interactions and similarity matrices.

Zhang et al. (2018) considered multiple characteristics of drugs to calculate similarity matrices. Next, they assumed that the matrices were multiples, projected the drugs onto the low-dimensional space of the interactive space, and introduced multiple normalizations. This group also proposed multi relational matrix factorization of DDI prediction, under which potential DDI are predicted by introducing multi relational matrix factorization of drug characteristics into the matrix factorization. Shtar et al. (2019) proposed prediction of DDI using adjacent matrix factorization (AMF) and adjacent matrix factorization propagation (AMFP). The authors used only known DDI as inputs to predict unknown DDI. AMF breaks down the matrix into an adjacent matrix of DDI, while AMFP (an extension of AMF) spreads potential factors from each drug to interacting drugs on an AMF basis.

Traditional approaches for predicting DDI can only predict their probability, not increases or decreases of drug efficacy during interaction. Yu et al. (2018) proposed a DDI-non-negative matrix factorization (DDINMF) approach to predict conventional and synthetic DDI based on semi-non-negative matrix factors. This approach can predict not only DDI, but also whether such interactions will enhance or decrease drug efficacy. DDINMF consists of a training stage and prediction stage. The authors expressed interactions in the DDI dataset as a symmetric interaction matrix, which was divided into basic and potential matrices using NMF. Shi et al. (2018) designed the triple matrix factorization (TMF)-based unified framework approach, which uses TMF to connect the adjacent matrix of the DDI network with the characteristic matrix of the drugs.

### Ensemble-Based Approach

The ensemble-based approach combines multiple approaches to predict unknown DDI. Zhang P et al. (2015) proposed that the computational burden of multi-label cases may be reduced by selecting appropriate information dimensions based on the mutual characteristics and side effects of drugs. Combined use of genetic algorithms and the multi-label k-nearest neighbor algorithm can define the optimal characteristic size and enables development of prediction models. A novel multi-label K-nearest adjacency method based on function selection (FS-MLKNN) is proposed, which can simultaneously determine key feature sizes and construct high-precision multi-label prediction models. FS-MLKNN takes two steps to establish the relationship between characteristic vectors and side effects. Firstly, information dimensions are selected by mutual information between functional dimensions and side effects to reduce the computational burden of multi-label learning. Then, genetic algorithm (GA) and multi-label K-nearest neighbor point method (MLKNN) were combined to determine the optimal feature size and develop a prediction model.

Zhang et al. (2017) built a prediction model based on various characteristics of drugs and known data about DDI according to neighbor-recommendation, random walk, and matrix disturbance approaches, which use flexible and diverse frameworks to combine different models with different ensemble rules. Deepika and Geetha (2018) predicted DDI through positive-unlabeled (PU) learning (Elkan and Keith, 2008) and meta-learning (Lemke et al., 2015), and proposed a learning framework for semi-supervised classifiers based on SVM. The PU-based classifier was used to generate meta-knowledge from the network, and the meta-classifier was designed to predict the probability of DDI from the generated meta-knowledge.

### Literature-based Approach

Literature-based prediction of DDI consists of two steps: first, extraction of the reasonable relationship between drugs from unstructured data sources (Vilar et al., 2018) (literature, electronic medical records, spontaneous reports, etc.) with a statistical or text-mining method, followed by use of natural language processing technique; second, prediction of unknown DDI from extracted information about the interactions between drugs using machine learning.

Tari et al. (2010) predicted DDI by combining text mining and reasoning. The process involved two stages: natural language extraction and reasoning. The authors used a parsing tree to extract various interactions and applied logical rules to predict interactions based on extracted interactions between new and existing drugs. Tatonetti et al. (2012b) divided FAERS into two sets of data: reports involving only one drug and reports involving two drugs, and constructed eight “clinically major” adverse event models. In each model, the drug information described was an introduction to the frequency of adverse events extracted from FAERS, and a logistic regression classifier was used to distinguish drugs that caused major clinical adverse events under study from those that did not; prediction was conducted based on the drug combination for each model. Kolchinsky et al. (2013) evaluated the performance of several classifiers, such as logistics regression, SVM, and discriminatory analysis, to distinguish relevant abstracts and PubMed articles containing evidence for pharmacodynamic DDI. Notably, their approach is also helpful to link causal mechanisms to potential DDI.

## Conclusion

The occurrence of DDI affects the treatment of patients and has become a serious problem for patient safety and drug management. The harm caused by DDI will be greatly reduced if machine learning can be used to efficiently predict DDI. To this end, it is urgent to develop better-performing machine learning approaches. This article describes existing machine learning-based approaches for predicting DDI. In the past 10 years, machine learning has been widely applied in bioinformatics and achieved good results. Under most of the existing approaches, drug similarity is taken as the most fundamental starting point for better prediction of DDI, assisted by a variety of other means. However, most current DDI predictions are limited to the interactions between two drugs. In the future work, we should not only pursue the accuracy of predicting the probability of drug-drug interactions, but also pursue the ability to accurately predict the types of drug-drug interactions. However, because the use of multiple drugs has become a trend in clinical medicine, it is urgent to develop methods to predict interactions between multiple drugs. It is our opinion that a number of excellent ways to solve this problem will be available in the near future.
